# Phosphatidylinositol-phospholipase C2 regulates pattern-triggered immunity in *Nicotiana benthamiana*

**DOI:** 10.1093/jxb/eraa233

**Published:** 2020-05-15

**Authors:** Akinori Kiba, Masahito Nakano, Miki Hosokawa, Ivan Galis, Hiroko Nakatani, Tomonori Shinya, Kouhei Ohnishi, Yasufumi Hikichi

**Affiliations:** 1 Laboratory of Plant Pathology and Biotechnology, Faculty of Agriculture, Kochi University, Nankoku, Kochi, Japan; 2 Okayama Prefectural Technology Center for Agriculture, Forestry, and Fisheries, 7549–1 Kibichuo-cho, Kaga-gun, Okayama, Japan; 3 Institute of Plant Science and Resources, Okayama University, Okayama, Japan; 4 Laboratory of Defense in Plant–Pathogen Interactions, Research Institute of Molecular Genetics, Kochi University, Nankoku, Kochi, Japan; 5 University of Edinburgh, UK

**Keywords:** Jasmonic acid, *Nicotiana benthamiana*, pathogen-associated molecular pattern-triggered immunity, phosphatidylinositol-phospholipase C2, *Ralstonia solanacearum*, virus-induced gene silencing

## Abstract

Phospholipid signaling plays an important role in plant immune responses against phytopathogenic bacteria in *Nicotiana benthamiana*. Here, we isolated two phospholipase C2 (PLC2) orthologs in the *N. benthamiana* genome, designated as PLC2-1 and 2-2. Both *NbPLC2-1* and *NbPLC2-2* were expressed in most tissues and were induced by infiltration with bacteria and flg22. *NbPLC2-1* and *NbPLC2-2* (*NbPLC2*s) double-silenced plants showed a moderately reduced growth phenotype. The induction of the hypersensitive response was not affected, but bacterial growth and the appearance of bacterial wilt were accelerated in *NbPLC2*s-silenced plants when they were challenged with a virulent strain of *Ralstonia solanacearum* that was compatible with *N. benthamiana*. *NbPLC2*s-silenced plants showed reduced expression levels of *NbPR-4*, a marker gene for jasmonic acid signaling, and decreased jasmonic acid and jasmonoyl-L-isoleucine contents after inoculation with *R. solanacearum*. The induction of pathogen-associated molecular pattern (PAMP)-triggered immunity (PTI) marker genes was reduced in *NbPLC2*s-silenced plants after infiltration with *R*. *solanacearum* or *Pseudomonas fluorescens*. Accordingly, the resistance induced by flg22 was compromised in *NbPLC2*s-silenced plants. In addition, the expression of flg22-induced PTI marker genes, the oxidative burst, stomatal closure, and callose deposition were all reduced in the silenced plants. Thus, NbPLC2s might have important roles in pre- and post-invasive defenses, namely in the induction of PTI.

## Teaser

Two phospholipase C2s in *Nicotiana benthamiana*, PLC2-1 and PLC2-2, complement each other during disease resistance responses and play important roles the induction of PAMP-triggered immunity.

## Introduction

Plants combat invading pathogens by employing a two-layered innate immune system (Thom ma *et al.*, 2011). The first layer is triggered upon perception of conserved molecular structures termed pathogen-associated molecular patterns (PAMPs) through pattern recognition receptors localized to the plasma membrane, and this is designated as PAMP-triggered immunity (PTI). Well-studied examples of PTI are Arabidopsis FLS2 and EFR, which recognize the bacterial flagellar component flg22 and the elongation factor thermo unstable (EF-Tu), respectively. Adapted pathogens have evolved a number of virulence mechanisms to suppress PTI by acquiring effector proteins (Bigeard *et al.*, 2015). As a counter-measure, plants have evolved the second layer of the innate immune system to directly or indirectly recognize effector proteins by their cognate resistance proteins, resulting in the initiation of effector-triggered immunity (ETI) (Gassmann and Bhattacharjee, 2012). PTI and ETI share signaling components that have distinct activation dynamics and amplitudes (Tsuda and Katagiri, 2010). Generally, PTI is characterized by broad-spectrum, transient, and relatively mild immune responses without an associated programmed cell death hypersensitive response (HR) (Segonzac *et al.*, 2011; Bigeard *et al.*, 2015). In contrast, ETI is characterized by specific, sustainable, and robust immune responses with HR (Jones and Dangl, 2006).

During PTI and ETI, plants trigger activation of diverse signaling cascades, such as generation of reactive oxygen species (ROS), spikes in cellular Ca^2+^, activation of MAP kinase, production of phytohormones, and transcriptional reprogramming. The most characterized Ca^2+^ spike results from an influx from the apoplast and endoplasmic reticulum that causes a rapid increase in the cytosolic concentration (Blume *et al.*, 2000; Lecourieux *et al.*, 2005). Plant cyclic nucleotide-gated ion channels provide a pathway for conductance of Ca^2+^ across the plasma membrane and thus facilitate the elevation of the cytosolic concentration. In Arabidopsis, cyclic nucleotide-gated ion channel 2 plays a pivotal role in allowing entry of Ca^2+^ into cells in response to pathogen signals (Ma and Berkowitz, 2011). The elevated cytosolic Ca^2+^ reportedly activates downstream intracellular signaling components. ROS generation is another event in immune signaling, and is mainly mediated by a respiratory-burst oxidase homologue (Rboh). ROS act not only as direct antimicrobial agents that cross-link components for cell-wall strengthening, but also as second messengers during immune signaling (Torres, 2010). In *Nicotiana benthamiana*, *NbRbohA* and *NbRbohB* are required for ROS generation to occur after treatment with hyphal cell wall components and the INF1 elicitin from *Phytophthora infestans* (Yoshioka *et al.*, 2003). The rapid activation of MAP kinase cascades is another important event in the downstream signal transduction during induction of PTI and ETI. In *N. tabacum* (tobacco) and *N. benthamiana*, protein kinases induced by salicylic acid (SA) and wounding are also activated rapidly after elicitation (Seo *et al.*, 1995; Lebrun-Garcia *et al.*, 1998; Zhang and Klessig, 1998; Dahan *et al.*, 2009).

Phospholipid-based signaling cascades are important for signal transduction in plant immune responses. Phospholipid turnover is mainly composed of cascades associated with diacylglycerol kinase and phospholipase C (PLC) and phospholipase D (PLD). Treatment with SA significantly increases the generation of phosphatidic acid (PA) by the activation of PLD (Rodas-Junco *et al.*, 2015). PLD is involved in defense signaling in non-host resistance against powdery mildew, and PLDδ may be the main participating isoform in Arabidopsis (Pinosa *et al.*, 2013). The silencing of diacylglycerol kinase cluster III abolishes PA production and strongly inhibits the ROS burst in tobacco in response to the elicitin cryptogein (Cacas *et al.*, 2017). We have previously identified the *SEC14* gene in *N. benthamiana*. Suppression of PLC and PLD activities, and production of diacylglycerol (DAG) and PA were observed in *NbSEC14-*silenced plants, resulting in compromised disease resistance against phytopathogenic bacteria through the jasmonic acid (JA)-dependent pathway (Kiba et al., 2012, 2014, 2016). Thus, phospholipid turnover may play an important role in the induction of immune responses in *N. benthamiana*.

PLCs are an important group of lipid-hydrolysing enzymes in both plants and animals. In plants, PLCs can be subdivided into the well-studied phosphatidylinositol-specific PLCs (PI-PLCs) and the recently identified phosphatidylcholine-PLCs (PC-PLCs). PI-PLCs act upon a specific substrate, PI (4,5) P_2_, at the glycerophosphate ester linkages of membrane phospholipids and lead to the generation of secondary messengers, such as DAG and inositol 1,4,5-trisphosphate (IP3) (Singh *et al.* 2015). A total of seven PLCs have been identified in tomato (Abd-El-Haliem *et al.*, 2016). We have searched the recently completed *N. benthamiana* genome sequence (https://solgenomics.net/) and identified 12 PLCs, and our objective here was to clarify their roles in plant immunity. We isolated two PLC2 orthologs, *NbPLC2-1* and *NbPLC2-2* (*NbPLC2*s), and determined the effects of silencing both on the induction of PTI and ETI in *N. benthamiana* by using several PTI-inducers and effectors, and by using *Pseudomonas syringae* pv. *Tabaci* and *Ralstonia solanacearum.* In the light of our results, we discuss the regulatory roles of NbPLC2s in immune responses in *N. benthamiana*.

## Materials and methods

### Biological and chemical materials

The flg22 peptide was obtained from the Funakoshi Co. Ltd. (Tokyo, Japan), and *Nicotiana benthamiana* was grown from seeds under a 16/8-h photoperiod in a growth room as described previously (Maimbo et al., 2007, 2010). *Pseudomonas fluorescens* 55 (an effective PTI inducer in *N. benthamiana*), *P. syringae* pv. *tabaci* 6605, the virulent compatible *Ralstonia solanacearum* strain OE1-1 (RsOE1-1), the avirulent *R. solanacearum* strain 8107, and *hrpY-*deficient RsOE1-1 that lacks the ability to deliver effectors into plant cells were cultured in peptone yeast-extract medium containing appropriate antibiotics as described previously (Chakravarthy *et al.*, 2010; Maimbo *et al.*, 2010; Ito et al., 2014*a*, 2014*b*). Based on previous reports, the bacterial population of *P. fluorescens* 55 was adjusted to 10^7^ (Chakravarthy *et al.*, 2010), *P. syringae* pv. *tabaci* 6605 to 10^4^ (Ito et al., 2014*a*, 2014*b*), RsOE1-1 and Rs8107 to 10^8^ (Maimbo *et al.*, 2010), and *hrpY-*deficient RsOE1-1 to 10^8^ (Kiba *et al.*, 2018). *Agrobacterium tumefaciens* was cultured in YEB medium (Maimbo *et al.*, 2010). The bacterial populations were determined by plating bacterial suspensions on *R. solanacearum* selective Hara-Ono medium plates at specified time-points (Maimbo *et al.*, 2010). The bacterial suspensions and the flg22 solution were infiltrated using syringes as described previously (Maimbo et al., 2007, 2010; Nguyen *et al.*, 2010). Control plants were infiltrated with water.

### RNA isolation and cDNA synthesis

Total RNAs were isolated from the stamens, gynoecium, petals, leaves, stems, petioles, and roots of 2-month-old *N. benthamiana* plants using NucleoSpin RNA Plant Kits (Macherey-Nagel), and qRT-PCR was completed according to the method described by Maimbo *et al.* (2007). A 1-µg sample of total RNA was used as the template for reverse-transcription using a ReverTra Ace^®^ qPCR RT Kit (Toyobo Co., Ltd.).

Sequence analysis was performed using the M4 and RV primers (Supplementary Table S1 at *JXB* online) with the reagents for the Big Dye Terminator Cycle Sequencing Kit (Applied Biosystems) and a 3100 Avant Automated Sequencer (Applied Biosystems) according to the manufacturer’s instructions. The sequence analysis was carried out using DNASIS (version 3.6; Hitachi) and the the BLAST network service from the NCBI (Altschul *et al.*, 1990).

### Virus-induced gene silencing

cDNA fragments for *NbPLC2-1*, *NbPLC2-2*, and the combined *NbPLC2-1* and *NbPLC2-2* sequences (*NbPLC2*s) were amplified using the primers listed in Supplementary Table S1 using *N. benthamiana* cDNA as the template. These cDNA fragments were independently subcloned into the TA cloning site of the pMD20 vector (TaKaRa Bio.) to create pMD-NbPLC2-1, pMD-NbPLC2-2, and pMD-NbPLC2s. These plasmids were digested with *Sal*I (TaKaRa Bio.) and ligated into *Sal*I-digested pPVX201 (Maimbo *et al.* 2007). The plasmids used for the virus-induced gene silencing (VIGS) experiments are listed in Supplementary Table S2. VIGS was conducted with pPVX201 containing the *NbPLC2-1*, *NbPLC2-2*, and combined *NbPLC2*s sequences. The cDNA fragments were amplified with the primers listed in Supplementary Table S1. The pPVX201 plasmid lacking any insert was used as a control, as previously described (Kiba *et al.*, 2012). These binary plasmids were transformed into *A. tumefaciens* strain GV3101 and inoculated into leaves of 4-week-old *N. benthamiana* as described previously (Nakano *et al.*, 2013). At 4 weeks after the initial inoculation, bacteria and flg22 were inoculated into a leaf located 3–4 leaves above the *Agrobacterium*-inoculated one as a challenge inoculation. The silencing efficiency was assessed by quantitative real-time PCR (qRT-PCR) assays (Supplementary Fig. S3).

### Bacterial population and disease index

Bacterial suspensions (10^8^ CFU ml^–1^) of RsOE1-1 and *hrpY*-deficient *R. solanacearum* were inoculated into *N. benthamiana* leaves. The bacterial populations after 24 h and 48 h were determined by plating on Hara-Ono plates. Plants inoculated with rsOE1-1 were labeled and inspected daily for wilting symptoms for 14 d. For each plant, the disease index on a scale of 0–4 was calculated as described previously (Maimbo *et al.*, 2010).

### Quantitative real-time PCR

Gene expression analysis was carried out using qRT-PCR and the ΔΔ*C*_T_ method as described by Maimbo *et al.* (2007). Briefly, the qRT-PCR was carried out in 20 µl of reaction mixture, containing 1 µl of cDNA template, 10 pM of the respective primers (Supplementary Table S1) and THUNDERBIRD qPCR MIX (Toyobo Co.), on an Applied Biosystems 7300 real-time PCR instrument. The cycling parameters were the same for all the primers: an initial 50 °C for 2 min and 95 °C for 10 min, followed by 40 cycles of 95 °C for 10 s and 60 °C for 1 min. Melting-curve runs were performed at the end of each PCR reaction to verify the specificity of the primers by the presence of a single amplification product. The relative quantification of gene expression was performed according to the manufacturer’s instructions using the comparative cycle threshold [Ct] method for the calculation of the Qty value. All values were normalized to the expression of the *actin* gene, which was used as an internal standard in each cDNA stock.

### Estimation of cell death

Cell death was measured by ion conductivity (Nakano *et al.*, 2013) using a Twin Cord B-173 conductivity meter (HORIBA, Kyoto, Japan).

### Phytohormone analysis

Phytohormone contents were determined using a method described previously by Kiba *et al.* (2012). Extracted samples were measured on a triple-quadrupole LC-MS/MS 6410 (Agilent Technologies) equipped with a Zorbax SB-C18 column (2.1 mm id×50 mm, 1.8 µm; Agilent Technologies). The amounts of hormones were calculated from the ratios of the endogenous hormone peaks and the known amounts of internal standards, and expressed in relation to the fresh weight of the samples used for extraction.

### Callose deposition assays

Callose deposition was detected using a modified version of a staining method using aniline blue (Ton and Mauch-Mani, 2004). Briefly, leaves of *N. benthamiana* were fixed and de-stained in 95% ethanol. The leaves were then washed with 0.07 M phosphate buffer (pH 9.0) and incubated for 1–2 h in 0.07 M phosphate buffer containing 0.01% aniline blue (Sigma). Samples were observed using a BX-51 epifluorescence microscope with a UV filter (Olympus). Callose deposition was quantified based on the number of deposits detected in digital photographs using the Photoshop Elements 7 software (Adobe Systems).

### PTI assays based on cell death

PTI assays based on cell death were conducted as described previously by Chakravarthy *et al.* (2010), with *P. fluorescens* 55 (1×10^9^ CFU ml^−1^) and *P. syringae* pv. *tabaci* 66455 (1×10^4^ CFU ml^−1^) as the inducer and challenger, respectively. The PTI was induced by infiltrating the leaves with the inducer, then 24 h later the challenger was applied to a partially overlapping area. Images were taken 5 d after the challenge inoculations.

### ROS measurements

ROS measurements were performed as described by Kobayashi *et al.* (2007). Leaves of *N. benthamiana* were infiltrated with 0.5 mM L-012 (Wako Pure Chemical Industries Ltd, Osaka, Japan) in 10 mM MOPS-KOH (pH 7.4) using a needleless syringe. Chemiluminescence was monitored continuously using a photon image processor equipped with a sensitive CCD camera (ARGUS-50 or Aquacosmos 2.5; Hamamatsu Photonics). Photons were integrally incorporated for 5 min after the treatment.

### Epidermal strip bioassays

Epidermal strip bioassays were carried out as described by Chen *et al.* (2004) with slight modifications. Leaves of *N. benthamiana* were incubated in MES buffer (10 mM MES-Tris, pH 6.0, containing 30 mM KCl and 0.1 mM CaCl_2_) for 90 min under light to open the stomata. The strips were then transferred to MES buffer in the absence or presence of 100 nM flg22 for 3 h and images of stomatal apertures were captured with an Olympus BX43 microscope. Measurements of 50 randomly selected stomata were taken. Each assay was repeated three times.

### Statistical analysis

Significant differences between means were determined using Student’s *t*-tests (two-sided).

## Results

### Identification of phosphatidylinositol phospholipase C2 from *Nicotiana benthamiana*

Based on PLC sequences from tomato (*Solanum lycopersicum* cv. Microtom), we searched for orthologs in *N. benthamiana* using the Sol Genomics Network (https://solgenomics.net/) and identified a total of 12. A phylogenic analysis of the amino acid sequences divided the NbPLCs into seven classes (Supplementary Fig. S1A). Two of them (Niben101Scf02221g00009 and Niben101Scf00318g03011) were classified into the same clade as SlPLC2; however, AtPLC2 belonged to a different clade. The deduced amino acid sequences of the full-length cDNAs of the PLC2 orthologs contained the PI-PLC-X, PI-PLC-Y, and PI3K-C2 domains, suggesting that they were phosphatidylinositol-specific PLCs (PI-PLCs; Supplementary Fig. S1B, C). We designated them as *NbPLC2-1* (Niben101Scf02221g00009) and *NbPLC2-2* (Niben101Scf00318g03011).

### Expression patterns of *NbPLC2-1* and *NbPLC2-2*

**Fig. 1.**
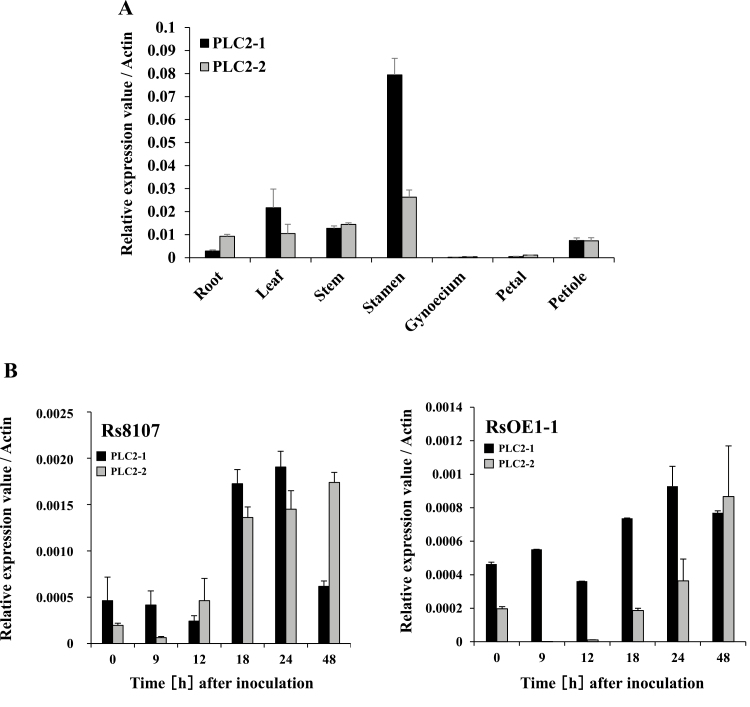
Expression patterns of *NbPLC2*s in *Nicotiana benthamiana*. (A) Relative expression of *NbPLC2-1* and *NbPLC2-2* in different tissues. (B) Relative expression of *NbPLC2-1* and *NbPLC2-2* in leaves of plants inoculated with avirulent *Ralstonia solanacearum* 8107 (Rs8107) or with virulent compatible *R. solanacearum* OE1-1 (RsOE1-1). Expression levels were determined by qRT-PCR and are relative to the *Actin* housekeeping gene. Data are means (±SD) of *n*=3 replicates.

Total RNAs were isolated from various organs of the plants, and qRT-PCR showed that *NbPLC2-1* and *NbPLC2-2* were expressed in all of them (Fig. 1A). The highest level of expression of *NbPLC2-1* was observed in the stamens, followed by the leaves, stems, petioles, roots, petals, and gynoecium. The highest level of expression of *NbPLC2-2* was also observed in the stamens, followed by the stems, leaves and roots equally, petioles, petals, and gynoecium.

To determine the expression profiles of *NbPLC2*s in response to inoculation with *R. solanacearum*, we used the virulent compatible strain OE1-1 (RsOE1-1) and the avirulent strain 8107 (Rs8107), and total RNAs were isolated from leaves at between 0–48 h after inoculation. Strong induction of both *NbPLC2-1* and *NbPLC2-2* was observed in leaves inoculated with Rs8107 (Fig. 1B), with the expression of *NbPLC2-1* showing a peak at 24 h whilst the highest expression of *NbPLC2-2* was observed at 48 h. The expression levels of *NbPLC2-1* and *NbPLC2-2* were lower in leaves inoculated with RsOE1-1 and increases in expression were not evident until 18–24 h after inoculation.

### Effects of silencing of *NbPLC2*s on resistance against *Ralstonia solanacearum*

To examine the roles of *NbPLC2*s in plant immunity, we carried out virus-induced gene silencing (VIGS). We created constructs for silencing both *PLC2*s (PLC2s-VIGS), and individually for *NbPLC2-1* (PLC2-1-VIGS) and *NbPLC2-2* (PLC2-2-VIGS) (Supplementary Fig. S2). We checked the specific suppression of *NbPLC2-1* and *NbPLC2-2* using qRT-PCR, which confirmed that the expression levels of both genes were reduced in plants inoculated with *Agrobacterium* carrying the NbPLC2s-VIGS construct and that the expression levels of the individual genes were reduced in the individual VIGS constructs (Supplementary Fig. S3A). It was notable that the expression of *NbPLC2-2* was increased in PLC2-1-VIGS plants and the expression of *NbPLC2-1* was increased in PLC2-2-VIGS plants. In terms of phenotype, compared with water-inoculated controls, PLC2s-VIGS plants showed a significant reduction in growth at 3 weeks after inoculation with the construct whilst no differences were observed in the PLC2-1-VIGS and PLC2-2-VIGS plants (Supplementary Fig. S3B, C).

**Fig. 2.**
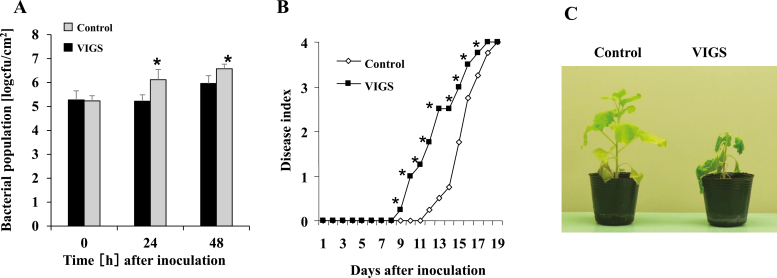
Responses of *NbPLC2*s-silenced *Nicotiana benthamiana* plants to virulent compatible strain of *Ralstonia solanacearum*. Leaves of plants at 8 weeks old were infiltrated with *R. solanacearum* OE1-1. Plants were silenced using VIGS. (A) Bacterial populations of *R. solanacearum* following inoculation. Data are means (±SD) of *n*=5 replicates. (B) Disease development of bacterial wilt according to a disease index scale of 0–4. Data are means of *n*=10 plants; for clarity, the error bars are not shown. (C) Images of control and VIGS plants at 12 d after inoculation. Significant differences between control and VIGS plants were determined using Student’s *t*-test: *P<0.05.

We then examined the roles of NbPLC2s using a *N. benthamiana–R. solanacearum* interaction model. We first inoculated PLC2s-VIGS, PLC2-1-VIGS, and PLC2-2-VIGS plants with the RsOE1-1 strain, a compatible pathogen that causes characteristic wilt symptoms in *N. benthamiana*. At 24 h after inoculation with RsOE1-1, the bacterial population was ~10-fold greater in PLC2s-VIGS plants compared with the control (Fig. 2A). Bacterial wilt was first observed in control plants at 12 d, and the plants were completely wilted at 18 d (Fig. 2B). In PLC2s-VIGS plants, accelerated wilting was observed, with symptoms first being visible at 9 d, and the plants were completely wilted by 17 d after inoculation (Fig. 2B, C). In contrast, no significant changes in disease development or bacterial populations were observed in the PLC2-1-VIGS and PLC2-2-VIGS plants (Supplementary Fig. S4). The results therefore indicated that NbPLC2s might play important roles in induced defense against *R. solanacearum* OE1-1, and that NbPLC2-1 and NbPLC2-2 might cooperatively regulate the responses. Another possibility was that activity of just one of the two PLC2s was sufficient to mediate the defense responses. Therefore, we used PLC2s-VIGS plants for further functional analysis of NbPLC2s in plant immunity.

Next, we inoculated plants with Rs8107, which is an incompatible pathogen that induces a hypersensitive response (HR) in *N. benthamiana*. HR lesions developed in both the control and PLC2s-VIGS plants 24 h after inoculation (Supplementary Fig. S5), and the magnitude of induced cell death and its timing were also similar, suggesting that the HR-mediated immune responses might have been independent of NbPLC2. Furthermore, silencing of the *NbPLC2*s had no effect on the induction of HR by *Agrobacterium*-mediated transient expression of the *R. solanacearum* effectors AvrA and PopP1 (Poueymiro *et al.*, 2009; Supplementary Fig. S5), and hence we concluded that NbPLC2 was not essential for the induction of effector-triggered immunity (ETI).

### Silencing of *NbPLC2*s reduces jasmonic acid-dependent defenses against *Ralstonia solanacearum*

**Fig. 3.**
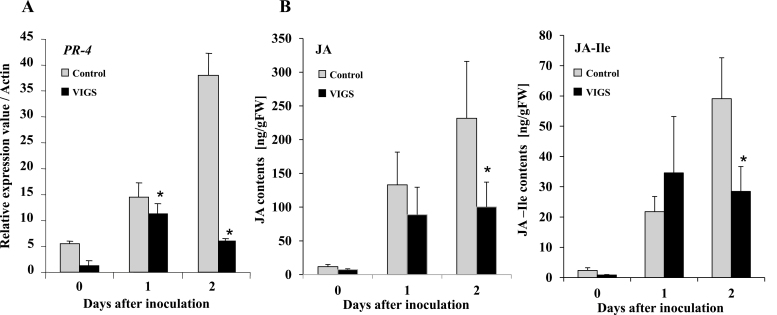
Suppression of jasmonic acid-dependent defense responses in *NbPLC2*s*-*silenced *Nicotiana benthamiana* plants inoculated with a virulent compatible strain of *Ralstonia solanacearum*. Leaves of plants at 8 weeks old were infiltrated with *R. solanacearum* OE1-1. Plants were silenced using VIGS. (A) Expression of *NbPR-4* (a marker gene for jasmonic acid signaling) was determined relative to that in the control and values were normalized against *Actin*. Data are means (±SD) of *n*=3 replicates. (B) Total contents of jasmonic acid (JA) and jasmonoyl-L-isoleucine (JA-Ile) were determined using LC-MS/MS. Data are means (±SD) of *n*=5 replicates. Significant differences between control and VIGS plants were determined using Student’s *t*-test: *P<0.05.

Phospholipid turnover plays an important role in defense responses against RsOE1-1 through JA signaling (Kiba *et al.*, 2012, Nakano *et al.*, 2013), and therefore we examined the effects of silencing *NbPLC2*s on JA signaling. Total RNA was extracted from leaves of control and PLC2s-VIGS plants at 0–2 d after inoculation with RsOE1-1. The expression level of *PR-4*, a marker gene for the JA signaling pathway, increased dramatically in control plants at both 1 d and 2 d after inoculation (Fig. 3A). In contrast, the increase in expression in PLC2s-VIGS plants at 1 d was significantly lower than in the control, and it had decreased at 2 d. Similar patterns were observed for the contents of JA and JA-L-isoleucine (Fig. 3B): whilst no significant differences were observed compared with the controls at 1 d after inoculation, the contents were both significantly reduced at 2 d in the PLC2s-VIGS plants. These results therefore suggested that NbPLC2s might be involved in JA-mediated immune responses against *R. solanacearum*.

### Stimulation of expression of *NbPLC2*s by PAMP-triggered immunity inducers

**Fig. 4.**
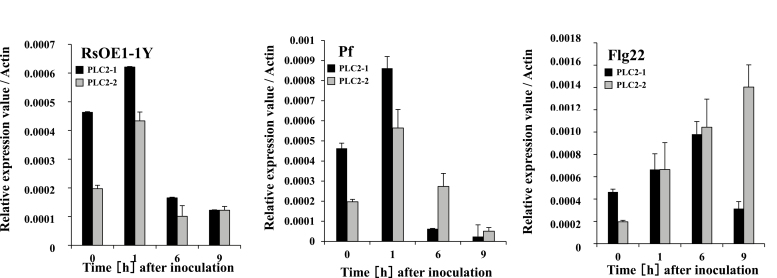
Induction of *NbPLC2-1* and *NbPLC2-2* in *Nicotiana benthamiana* by pathogen-associated molecular pattern (PAMP)-triggered immunity inducers. Plants were inoculated with either a *hrpY*-deficient mutant of virulent compatible *Ralstonia solanacearum* OE1-1 (RsOE1-1Y), *Pseudomonas fluorescens* (Pf), or 100nM flg22 peptide (Flg22). Expression levels of *NbPLC2-1* and *NbPLC2-2* were determined by qRT-PCR and are relative to the control, with values normalized against *Actin*. Data are means (±SD) of *n*=3 replicates.

The silencing of *NbPLC2*s essentially had no effect on the induction of HR by the incompatible Rs8107 strain and by type III effectors from *R. solanacearum*. However, PLC2s-VIGS plants showed a reduced-resistance phenotype against the compatible RsOE1-1 strain. Jones and Dangl (2006) defined resistance activated by virulent pathogens on susceptible hosts as basal disease resistance. Accordingly, it can be described as PAMP-triggered immunity (PTI) plus weak ETI minus effector-triggered susceptibility. Basal disease resistance is then mainly covered by PTI (Jones and Dangle, 2006). We therefore examined the correlation between *NbPLC2*s and PTI by first determining their expression patterns in the presence of PTI inducers. We used a type III secretion system (*hrpY*)-deficient mutant of RsOE1-1 that lacks the ability to deliver effectors into plant cells and induces PTI in *N. benthamiana*, and we also used *Pseudomonas fluorescens* and the bacterial flagellar component flg22, which are effective PTI inducers in *N. benthamiana* (Chakravarthy *et al.*, 2010). Strong inductions of *NbPLC2-1* and *NbPLC2-2* were observed 1 h after inoculation with the *hrp*-deficient mutant and 1 h after inoculation with *P. fluorescens* (Fig. 4). Inoculation with flg22 induced increasing expression of *NbPLC2-1* from 1–6 h, after which it declined, whilst expression of *NbPLC2-2* increased from 1–9 h after treatment. *NbPLC2*s thus appeared to be expressed after the perception of PAMPs.

### Silencing of *NbPLC2* reduces PAMP-triggered immune responses to *Ralstonia solanacearum*

**Fig. 5.**
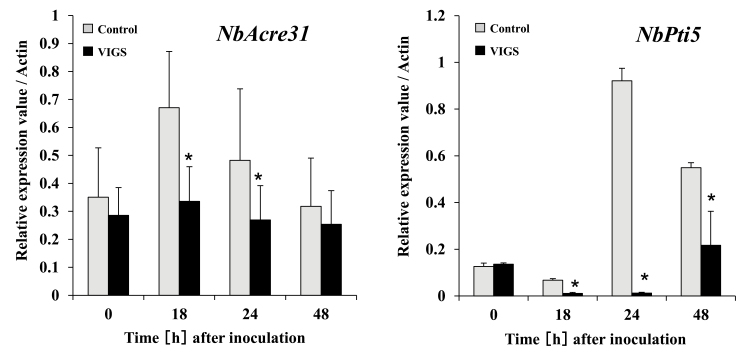
Suppression of pathogen-associated molecular pattern (PAMP)-triggered immunity (PTI) in *Nicotiana benthamiana NbPLC2*s*-*silenced plants inoculated with a virulent compatible strain of *Ralstonia solanacearum*. Leaves of plants at 8 weeks old were infiltrated with *R. solanacearum* OE1-1. Plants were silenced using VIGS. Expression of the PTI marker genes *NbAcre31* and *NbPti5* were determined by qRT-PCR and are relative to the control, with values normalized against *Actin*. Data are means (±SD) of *n*= 3 replicates. Significant differences between control and VIGS plants were determined using Student’s *t*-test: *P<0.05.

Because the expression levels of *NbPLC2-1* and *NbPLC2-2* were induced by the *hrp*-deficient mutant of RsOE1-1, by *P. fluorescens*, and by flg22, we hypothesized that NbPLC2s may be involved in the initiation of PTI. To assess the effects of silencing of *NbPLC2*s on the induction of PTI, we examined the expression levels of the PTI marker genes *NbAcre31* and *NbPti5* after inoculation with RsOE1-1. In control plants, increased expression of *NbAcre31* was observed between 18–24 h after inoculation and increased expression of *NbPti5* was observed between 24–48 h after inoculation (Fig. 5). In agreement with our hypothesis, the expression levels of both genes were significantly reduced in PLC2s-VIGS plants relative to the controls following inoculation.

**Fig. 6.**
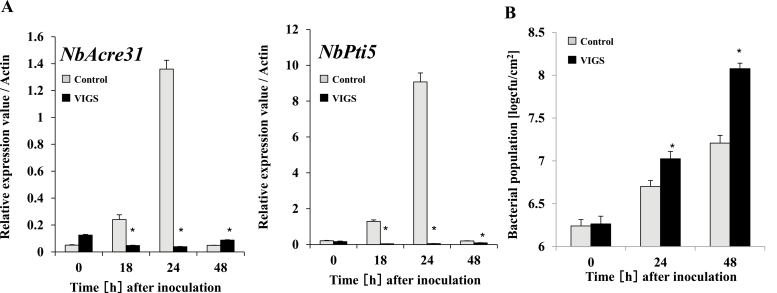
Effects of silencing *NbPLC2*s in *Nicotiana benthamiana* on pathogen-associated molecular pattern (PAMP)-triggered immunity (PTI) in response to a *hrpY*-deficient mutant of *Ralstonia solanacearum*. Leaves of plants at 8 weeks d old were infiltrated with the *R. solanacearum* mutant. Plants were silenced using VIGS. (A) Expression of the PTI marker genes *NbAcre31* and *NbPti5* were determined by qRT-PCR and are relative to the control, with values normalized against *Actin*. (B) Bacterial populations of the *R. solanacearum* mutant following inoculation. Data are means (±SD) of *n*=5 replicates. Significant differences between control and VIGS plants were determined using Student’s *t*-test: *P<0.05.

We then inoculated plants with the *hrp*-deficient mutant of RsOE1-1 and observed increased expression of *NbAcre31* and *NbPti5* after 18–24 h in the controls, whereas expression in the PLC2s-VIGS plants was significantly lower at these time-points (Fig. 6A). To further confirm their role in PTI, we determined the effects of *NbPLC2*-silencing on the growth of the *hrp*-deficient mutant. Bacterial growth increased following inoculation in both control and PLC2s-VIGS plants, but the rate was higher in the latter with the result that populations were ~10-fold greater after 48 h in the silenced plants (Fig. 6B). These results further supported the involvement of NbPLC2s in the induction of PTI.

### Silencing of *NbPLC2*s reduces PAMP-triggered immune responses to *Pseudomonas fluorescens*

**Fig. 7.**
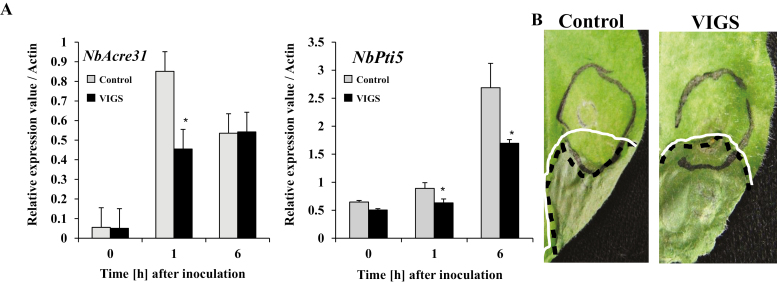
Effect of *NbPLC2*s-silencing in *Nicotiana benthamiana* on pathogen-associated molecular pattern (PAMP)-triggered immunity (PTI) in response to *Pseudomonas fluorescens* (an effective PTI inducer). Leaves of plants at 8 weeks old were infiltrated with *P. fluorescens*. Plants were silenced using VIGS. (A) Expression of the PTI marker genes *NbAcre31* and *NbPti5* were determined by qRT-PCR and are relative to the control, with values normalized against *Actin*. Data are means (±SD) of *n*=3 replicates. Significant differences between control and VIGS plants were determined using Student’s *t*-test: *P<0.05. (B) A cell death-based assay for PTI. *Pseudomonas syringae* pv. *tabaci* was used as the death-inducible challenger, and *P. fluorescens* was infiltrated into the leaves to induce PTI (area within grey circle). At 7 h after *P. fluorescens* inoculation, the same leaves were challenged with *P. syringae* pv. *tabaci* (area within the white line). The area within the black dotted line indicates the necrotic lesions caused by *P. syringae* pv. *tabaci*. The images were taken 5 d after inoculation with *P. syringae* pv. *tabaci*.

*Pseudomonas fluorescens* is an effective PTI inducer in *N. benthamiana* (Chakravarthy *et al.*, 2010), and so we used it to inoculate control and *NbPLC2*-silenced plants to induce responses. Expression of *NbAcre31* increased after 1 h following inoculation in both control and PLC2s-VIGS plants, but it was significantly higher in the control (Fig. 7A). The levels were then the same after 6 h. For *NbPti5*, expression increased at 1 h and 6 h after inoculation and at both time-points it was significantly higher in the controls than in the silenced plants.

An assay based on cell death using *P. fluorescens* has been reported to be an effective tool for examining PTI responses (Oh and Collmer, 2005). We used *P. fluorescens* as a PTI inducer and *P. syringae* pv. *tabaci* as a challenger. In the control and PLC2s-VIGS plants, infection with *P. syringae* pv. *tabaci* resulted in necrotic lesions in leaf regions in which *P. fluorescens* was not infiltrated (Fig. 7B). In control plants, necrotic lesions were suppressed in the region in which *P. fluorescens* and *P. syringae* pv. *tabaci* overlapped, indicating that PTI was effectively induced. In contrast, necrotic lesions were observed in the overlapping area of PLC2s-VIGS plants inoculated with both bacteria. This suggested that the silencing of the *NbPLC2*s impaired the PTI response by *P. fluorescens*.

### Silencing of *NbPLC2*s reduces PAMP-triggered immune responses to flg22

**Fig. 8.**
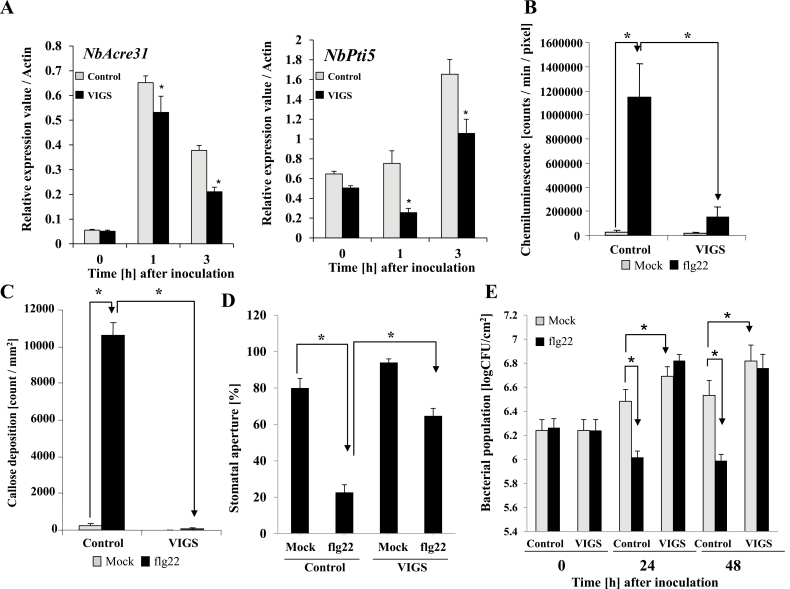
Effects of *NbPLC2*s-silencing in *Nicotiana benthamiana* on pathogen-associated molecular pattern (PAMP)-triggered immunity (PTI) induced by flg22. Flg22 (100 nM) was used as the PTI-inducer and infiltrated into leaves of 8-week -old plants. Plants were silenced using VIGS. (A) Expression of the PTI marker genes *NbAcre31* and *NbPti5* were determined by qRT-PCR and are relative to the control, with values normalized against *Actin*. Data are means (±SD) of *n*=3 replicates. (B) Levels of reactive oxygen species determined 30 min after the flg22 treatment. Chemiluminescence intensities mediated by L-012 were quantified using a photon image processor. Photons were integrally incorporated for 5 min after the flg22 treatment. Data are means (±SD) of *n*=4 replicates. (C) Callose deposition at 24 h after flg22 treatment, as determined by staining with aniline blue. Fluorescent deposits were visualized using fluorescence microscopy. Data are means (±SD) of *n*=5 replicates. (D) Stomatal apertures in the leaves at 3 h after treatment with flg22. Data are means (±SD) of *n*=50 replicates from three independent experiments. (E) Flg22 was infiltrated into the leaves of control and *NbPLC2s*-silenced plants. After 24 h, a virulent compatible strain of *Ralstonia solanacearum*, OE1-1, was inoculated as a challenger into the flg22-infiltrated area, and the bacterial populations were determined at subsequent time-points. Data are means (±SD) of *n*=5 replicates. Significant differences between means were determined using Student’s *t*-test: *P<0.05.

In both control and PLC2s-VIGS plants, a strong increase in expression of *NbAcre31* was observed 1 h after treatment with the flg22 elicitor, and expression remained elevated at 3 h (Fig. 8A); however, at both time-points the expression in the silenced plants was significantly lower than in the controls. For *NbPti5*, the highest levels of expression were observed after 3 h, but again at both time-points the expression in the silenced plants was significantly lower than in the controls.

ROS generation is another hallmark of a PTI response, and we found that it was dramatically induced 30 min after the inoculation with flg22 in the control plants (Fig. 8B). Whilst an increase in ROS was also observed in PLC2s-VIGS plants, the response was much less than in the control plants.

Callose deposition occurs during PTI responses to counteract pathogen invasions. In control plants, we found a large increase in callose deposition 24 h after the inoculation with flg22 while PLC2s-VIGS plants showed no response (Fig. 8C, Supplementary Fig. S6A).

Guard cells exhibit innate immune responses to pathogens and PAMP compounds, such as flg22, which induce stomatal closure (Zhang *et al.*, 2009). At 3 h following treatment with flg22, the size of the stomatal apertures was clearly reduced in both control and PLC2s-VIGS plants, suggesting induced closure (Fig 8D, Supplementary Fig. S6B). However, the induced closure was significantly less in PLC2s-VIGS plants relative to the control.

We then treated leaves with flg22, and 24 h later inoculated the treated areas with *R. solanacearum* strain OE1-1. The bacterial population increased at 24 h and 48 h after inoculation with RsOE1-1 in control plants, and populations were greater in PLC2s-VIGS plants than in the controls (Fig. 8E; see also Fig. 2A). The proliferation of bacteria was suppressed by pre-treatment with flg22 in the control plants, indicating that it effectively induced PTI against RsOE1-1. In contrast, no suppression of bacterial growth by flg22 was observed in the PLC2s-VIGS plants.

## Discussion

Phospholipase Cs (PLCs) represent an important group of lipid-hydrolysing enzymes in both plants and animals (Pokotylo *et al.*, 2014). In plants, phosphatidylinositol-specific (PI-)PLCs act on a specific substrate, PI (4,5) P_2_, at glycerophosphate ester linkages of membrane phospholipids and lead to the generation of secondary messengers, such as DAG and IP3. The phosphorylated products of DAG and IP3, namely PA, diacylglycerol pyrophosphate, and hexakisphosphate may function as second messengers in plants (van Leeuwen *et al.*, 2007, Xue *et al.*, 2007). Plant PI-PLCs have been implicated in a number of cellular processes and signal transduction events during differentiation and development. In Arabidopsis, nine PI-PLCs (PLC1–9) have been identified (Tasma *et al.*, 2008). More recently, PLCs have been detected in the tomato genome and classified into seven groups (Abd-El-Haliem *et al.*, 2016). In our current study, we found 12 PLC orthologs in *N. benthamiana* (Supplementary Fig. S1). Based on a phylogenic analysis of the amino acid sequences, we designated two PLC orthologs, Niben101Scf02221g00009 and Niben101Scf00318g03011, as NbPLC2-1 and NbPLC2-2, respectively. Intriguingly, AtPLC2 belonged to a different clade from NbPLC2-1, NbPLC2-2, and SlPLC2 (Supplementary Fig. S1A; Abd-El-Haliem *et al.*, 2016). In addition, 15 amino acids were lacking in the central regions of NbPLC2-1, NbPLC2-2, and SlPLC2 (Supplementary Fig. S1B). Hence, the structures of PLC2s from the Solanaceae were different from those of the Brassicaceae, and these differences might be correlated with functional differences.

*PLC1* and *PLC3* are expressed in Arabidopsis pollen and have significant roles in pollen-tube growth (Dowd *et al.*, 2006, Helling *et al.*, 2006). PI-PLC has also been found to play a significant role during the process of asymmetric cell division that generates stomatal complexes in maize (Apostolakos *et al.*, 2008). In petunia, the catalytically inactive form of PLC1 competes with the native form, and this results in an alteration of the Ca^2+^ gradient and reorganization of the cytoskeleton, leading to delocalized growth and swollen tips in pollen tubes (Dowd *et al.*, 2006). Among the nine Arabidopsis PLCs, only PLC2 is expressed dominantly and ubiquitously in most tissues (Hirayama *et al.*, 1997; Li *et al.*, 2015) and it is the primary phospholipase in phosphoinositide metabolism (Kanehara *et al.*, 2015). Disruption of PLC2 can lead to sterility because the development of both male and female gametophytes is severely perturbed in homozygous *plc2* Arabidopsis mutants (Li *et al.*, 2015; Di Fino *et al.*, 2017). PLC2 also functions in auxin‐modulated root development in Arabidopsis (Chen *et al.*, 2019). Thus, plant PI-PLCs may act as important regulators of various signaling pathways in different processes of growth and development. In our study, expression of *NbPLC2-1* and *NbPLC2-2* was observed in the stamen, gynoecium, petals, leaves, stems, and roots (Fig. 1A). *NbPLC2*s-silenced plants displayed phenotypes with moderately retarded growth relative to controls, with ~40% reduced height (Supplementary Fig. S3B, C). However, there were no phenotypic changes in plants with *NbPLC2-1* or *NbPLC2-2* silenced individually. Intriguingly, the expression level of *NbPLC2-2* was significantly increased in *NbPLC2-1-*silenced plants, whilst the expression of *NbPLC2-1* was significantly increased in *NbPLC2-2*-sielnced plants. Taken together, our results indicate that NbPLC2s appear to be involved in growth and development in *N. benthamiana* plants, at least in the stages following the initiation of VIGS in our experiments. In addition, NbPLC2-1 and NbPLC2-2 may mutually complement each other at the transcriptional level during these developmental stages.

Several PLCs participate in abiotic stress responses. The overexpression of maize and tobacco PLCs confers higher drought and salt tolerance levels in transgenic plants (Wang *et al.*, 2008, Tripathy *et al.*, 2012). In Arabidopsis, PI-PLCs have been implicated in the accumulation of the osmolyte proline that leads to adaptive responses following ionic hyperosmotic stress (Parre *et al.*, 2007). A PLC-mediated signal transduction pathway is also induced during cold stress in plants (Ruelland *et al.*, 2002). PLCs may also have significant roles in responses to heat stress. For example, PI-PLC proteins accumulate in pea plants after heat-stress treatments (Liu *et al.*, 2006; Ruelland and Zachowski, 2010). Arabidopsis PLC9 has been implicated in heat-stress responses, and the *atplc9* mutant exhibits a highly thermosensitive phenotype. Accumulation of HSP18.2 and HSP25.3 is reduced in *atplc9* and enhanced in *AtPLC9*-overexpressing lines after exposure to heat stress (Zheng *et al.*, 2012). An important role for AtPLC3 in thermo-tolerance has been established in Arabidopsis through a reduction in the heat-induced levels of Ca^2+^ in *atplc3* plants (Gao *et al.*, 2014). In rice, PLC1-mediated Ca^2+^ signaling is essential for controlling the accumulation of Na^+^ that leads to salt tolerance (Li *et al.*, 2017). In contrast, AtPLC4 negatively regulates the salt tolerance of Arabidopsis seedlings through Ca^2+^ regulatory processes (Xia *et al.*, 2017). PLC2 is involved in stress responses related to the endoplasmic reticulum in Arabidopsis (Kanehara *et al.*, 2015). It remains to be determined whether the NbPLC2s isolated in our study may also participate in abiotic stress responses.

As well as abiotic stress responses, PLCs are also involved in responses to biotic stress, including plant immunity (Canonne *et al.*, 2011). The PI-PLC family is required for HR-mediated defense responses and induction of effector-triggered immunity (ETI). Phytoalexin and ROS production, together with the HR, are reduced by the PI-PLC inhibitor U73122 in riboflavin- and Cf-4/Avr4-elicited tobacco cells (de Jong *et al.*, 2004; Wang *et al.*, 2013). Silencing of *SlPLC6* results in a reduction of HR and increased colonization of Avr4-carriyng *Cladosporium fulvum* in *Cf-4* containing tomato plants (Vossen *et al.*, 2010). Avr4-induced HR is also reduced in the resistance *Cf-4* carrying tomato after *SlPLC4*-silencing. In addition, the heterologous expression of *SlPLC4* results in accelerated Avr4/Cf-4-induced HR in *N. benthamiana* (Abd-El-Haliem *et al.*, 2016). Treatment of tomato suspension cells with HR-inducible fungal xylanase leads to a rapid increase in nitric oxide, which is responsible for PI-PLC activity and consequent defense responses (Raho *et al.*, 2011). The production of nitric oxide and induction of HR are required to increase the expression levels of *SlPLC5* in the xylanase-treated cells. Thus, numerous examples show that induction of HR and ETI are dependent on the functions of PI-PLC family members in tomato, and possibly in other plants. SlPLC2 is also required for HR, as shown by the expression of *hsr203J* being suppressed in *SlPLC2*-silenced plants (Gonorazky *et al.*, 2014). Our results showed that the NbPLC2s were also required for ROS production (Fig. 8B), similar to SlPLC2. In contrast, the silencing of the NbPLC2s did not affect ETI, including induction of HR (Supplementary Fig. S5A, B). Thus, the NbPLC2s did not appear to be required for ETI responses.

SlPLC2 is also required for the xylanase-induced expression of defense-related genes. Reduced expression of the SA-dependent *PR-1a* gene is observed in *SlPLC2*-silenced plants, indicating it has a role in SA signaling (Gonorazky *et al.*, 2014). SlPLC2 is also required for plant susceptibility against *Botrytis cinerea* through the competitive suppression of JA-dependent defenses caused by up-regulation of the SA-signaling pathway (Gonorazky *et al.*, 2016). In contrast, silencing of *NbPLC2*s affected JA-related defenses (Fig. 3), and hence they may have a role in JA-dependent defense responses in *N. benthamiana*.

In addition to HR-mediated defenses and ETI, PLCs play important roles in defenses without HR, including induction of PTI. Both the flagellin-triggered response and the internalization of the corresponding receptor, FLS2, in Arabidopsis are suppressed by the inhibition of PLC activity (Abd-El-Haliem *et al.*, 2016). *PLC2*-silenced Arabidopsis plants are susceptible to a spray treatment of the type-III secretion system-deficient bacterial strain *P. syringae* pv. *tomato* DC3000 (*hrc*C–) but not to the wild-type *P. syringae* pv. *tomato* DC3000 (D’Ambrosio *et al.*, 2017). In contrast, PLC2 does not affect bacterial growth when the bacteria are syringe-infiltrated into the apoplast. In response to flg22, *PLC2*-silenced Arabidopsis show reduced stomatal closures. Thus, PLC2s may control stomatal pre-invasive, but not post-invasive, immunity. In our present study, expression of *NbPLC2-1* and *NbPLC2-2* were induced by PTI inducers, such as *hrp*-deficient *R. solanacearum*, *P. fluorescens*, and flg22 (Fig. 4); however, intriguingly, the induction patterns were different. Therefore, we speculate that NbPLC2-1 and NbPLC2-2 might have different roles during the immune responses. Silencing the *NbPLC2*s negatively affected the expression of PTI reporter genes when control and *NbPLC2*-silenced plants were infiltrated with wild-type *R. solanacearum*, *hrp*-deficient *R. solanacearum*, *P. fluorescens*, or flg22 (Figs 3–7). The suppression of PTI induction was observed using a cell death-based assay with *P. fluorescens* and *P. syringae* pv. *tabaci.* Flg22-induced callose deposition and hence disease resistance were also compromised in *NbPLC2*s-silenced plants (Fig. 8C, Supplementary Fig. S6A). These results collectively support a significant role for PLC2 in PTI responses. In addition, flg22-induced stomatal closure was reduced by silencing of the *NbPLC2*s (Fig. 8D, Supplementary Fig. S6B). Furthermore, enhanced bacterial growth occurred in *NbPLC2*s-silenced plants when either *R. solanacearum* or a *hrpY* mutant were infiltrated into the apoplast area. These results suggest that NbPLC2s control not only stomatal pre-invasive, but also post-invasive, immunity in *N. benthamiana*.

In summary, we have demonstrated that NbPLC2s contribute to the induction of pre- and post-invasive PTI responses in *N. benthamiana*. While undergoing PTI induction, NbPLC2s may be activating JA and JA-mediated immune responses, leading to the suppression of bacterial infections. These results provide novel insights into the roles of PLC2 and the PLC family in the regulation of plant immunity. Further studies are necessary to clarify the complex mechanisms by which the NbPLC2 protein is engaged in PTI responses, and to characterize the phospholipid turnover involved in the PTI-signaling cascade.

## Supplementary data

Supplementary data are available at *JXB* online.

Fig. S1. Characterization of the phosphatidylinositol-specific phospholipase C in *Nicotiana benthamiana*.

Fig. S2. Nucleotide sequences of *NbPLC2-1* and *NbPLC2-2*.

Fig. S3. Phenotypes of *NbPLC2*s-, *NbPLC2-1*-, and *NbPLC2-2*-silenced plants.

Fig. S4. Responses of *NbPLC2*s-, *NbPLC2-1*-, and *NbPLC2-2*-silenced plants to compatible *Ralstonia solanacearum*.

Fig. S5. Effects of *NbPLC2*s-silencing on the induction of the hypersensitive response by effectors from incompatible *Ralstonia solanacearum*.

Fig. S6. Callose deposition and stomatal closure in *NbPLC2*s-silenced plants.

Table S1. Primers used in this study.

Table S2. Plasmids used in this study.

eraa233_suppl_Supplementary_Material

## Abbreviations

HR: hypersensitive response
hrp: hypersensitive response and pathogenicity
PAMPs: pathogen-associated molecular patterns
PLC: phospholipase C
PTI: PAMPtriggered immunity
Rs: Ralstonia solanacearum
VIGS: virus-induced gene silencing
