# Supplementary material for: Phosphatidylinositol-phospholipase C2 regulates pattern-triggered immunity in Nicotiana benthamiana
Source: J Exp Bot. 2020 May 15;71(16):5027–38. doi: 10.1093/jxb/eraa233 (PMC7410187; doi:10.1093/jxb/eraa233)
Supplement: eraa233_suppl_Supplementary_Material [file eraa233_suppl_supplementary_material.pdf]

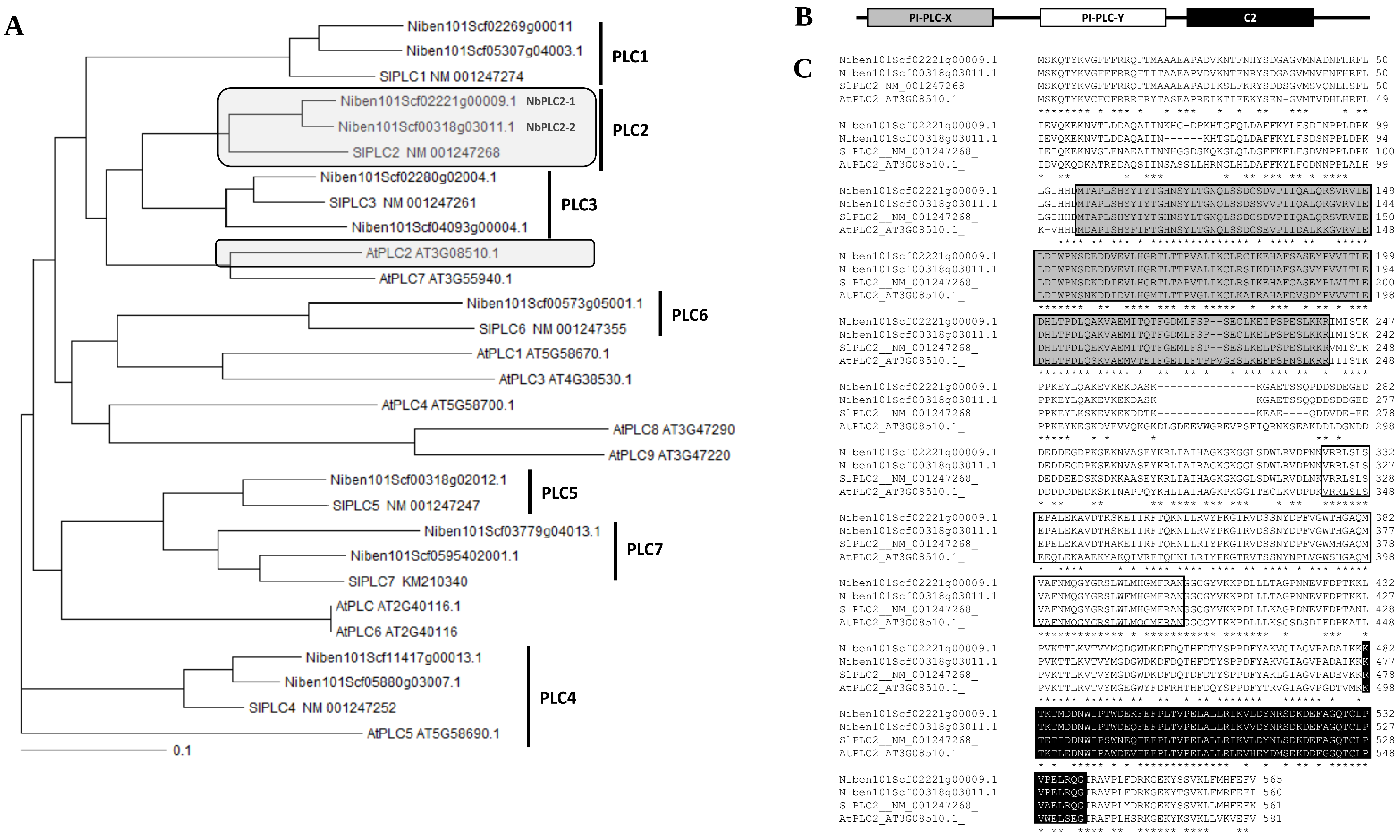

Figure S1 Characterization of the phosphatidylinositol-specific phospholipase C in *Nicotiana benthamiana*

(*A*) Phylogenic tree of phosphatidylinositol-specific phospholipase C based on amino acid sequence from *Nicotiana benthamiana* and *Solanum lycopersicum* cv. Microtom. (B) Schematic representation of the PLC2 protein structure. PI-PLC-X and PI-PLC-Y are the conserved X and Y domain essential for PLC activity, respectively. C2 is the calcium-dependent lipid-binding domain. (C) Alignment of deduced amino acid sequence of *NbPLC2-1* (Niben101Scf02221g00009), *NbPLC2-1* (Niben101Scf00318g03011), *SlPLC2* (NM_001247268) and *AtPLC2* (AT3G08510.1). Asterisks showed identical amino acids. Gray box and white boxes show PI-PLC-X and PI-PLC-Y domain, respectively. The C2 domain was shown in black box.

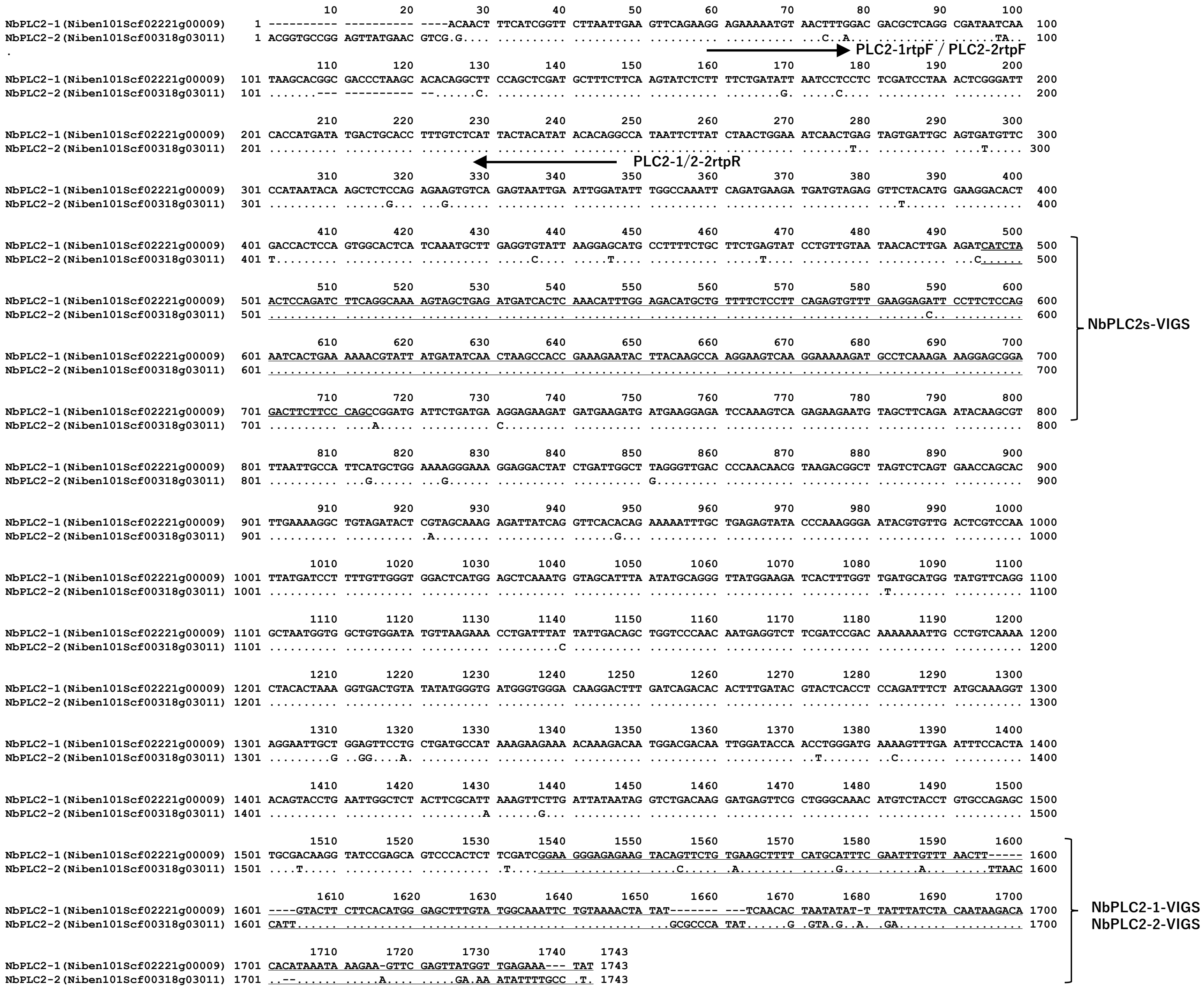

Figure S2 Nucleotide sequence of *NbPLC2-1* and *NbPLC2-2*

Alignment of nucleotide sequence of *NbPLC2-1* (Niben101Scf02221g00009) and *NbPLC2-1* (Niben101Scf00318g03011). Nucleotide sequence used for virus-induced gene silencing were underlined (NbPLC2s-VIGS, NbPLC2-1-VIGS, or NbPLC2-2-VIGS). Arrows indicated nucleotide sequence used for qRT-PCR (PLC2-1/2-2rtpF, PLC2-1rtpR and PLC2-2rtpR).

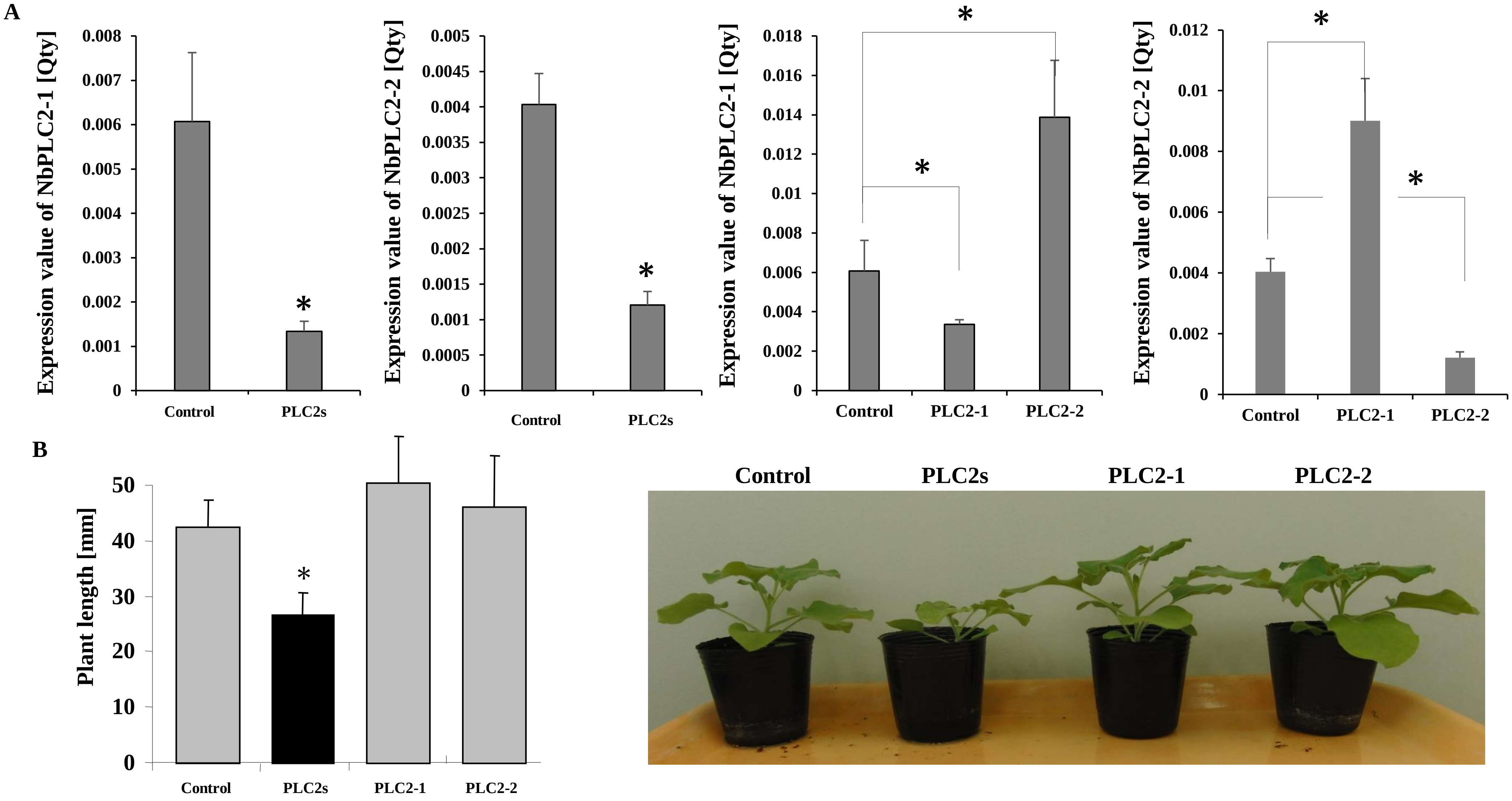

Figure S3 Phenotypic observation of *NbPLC2s*, *NbPLC2-1* and *NbPLC2-2*-silenced plants.

(A) Total RNA was isolated from control (Control), *NbPLC2s*-silenced (PLC2s), *NbPLC2-1*-silenced (PLC2-1) and *NbPLC2-2*-silenced (PLC2-2) leaves. Expression values were normalized against actin gene expression levels. Data represent the means and standard deviations from triplicate experiments. (B) Plant length was determined 3 weeks after inoculation with *Agrobacterium tumefaciens*. (C) Photograph was taken 3 weeks after inoculation with *Agrobacterium tumefaciens*. Values represent mean plant length (n = 7) with SD. Asterisks denote values significantly different from those of water-inoculated controls (*; $P < 0.05$, t-test).

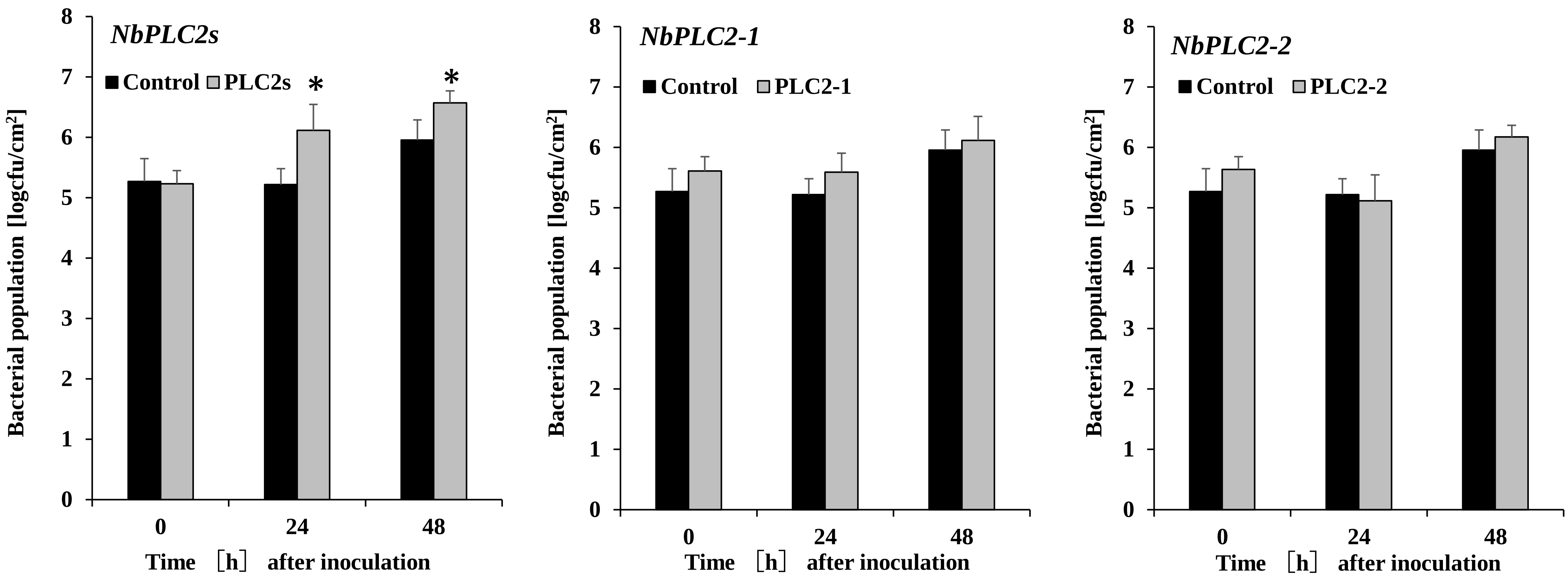

Figure S4 Response of *NbPLC2s, NbPLC2-1* and *NbPLC2-2*-silenced plants against compatible *R. solanacearum*.

Control and *NbPLC2s, NbPLC2-1* and *NbPLC2-2*-silenced plant leaves infiltrated with *R. solanacearum* OE1-1. Bacterial population of *R. solanacearum* OE1-1 was determined by plating at specified time points. Values are means of five replicate experiments with SD. Asterisks denote values significantly different from those of control plants (*; P < 0.05, *t*-test).

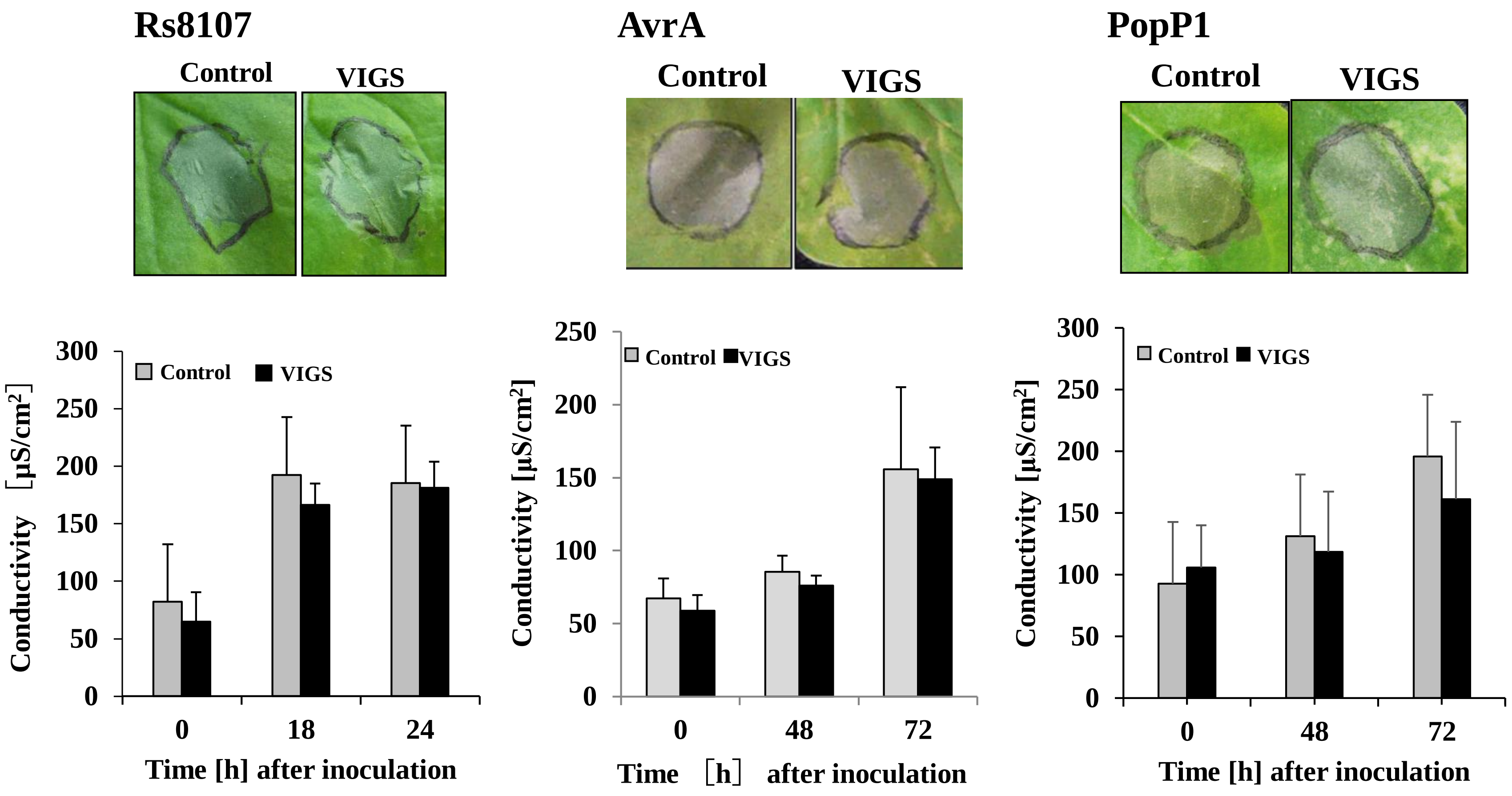

Figure S5 Effect of *NbPLC2s*-silencing on HR induction by effectors from incompatible *R. solanacearum*.

Control and *NbPLC2s*-silenced (VIGS) *N. benthamiana* plants were infiltrated with *R. solanacearum* 8107 (Rs8107), HR-inducible *Agrobacterium* harboring 35S-GUS (control GUS), 35S-AvrA (AvrA) and 35S-PopP1 (PopP1). (A) Pictures of *N. benthamiana* leaves taken 1 or 4 days after infiltration with *R. solanacearum* 8107 or *Agrobacterium*, respectively. (B) Cell death was determined by measurement of ion conductivity (leakage). Values represent mean with SD (n=5).

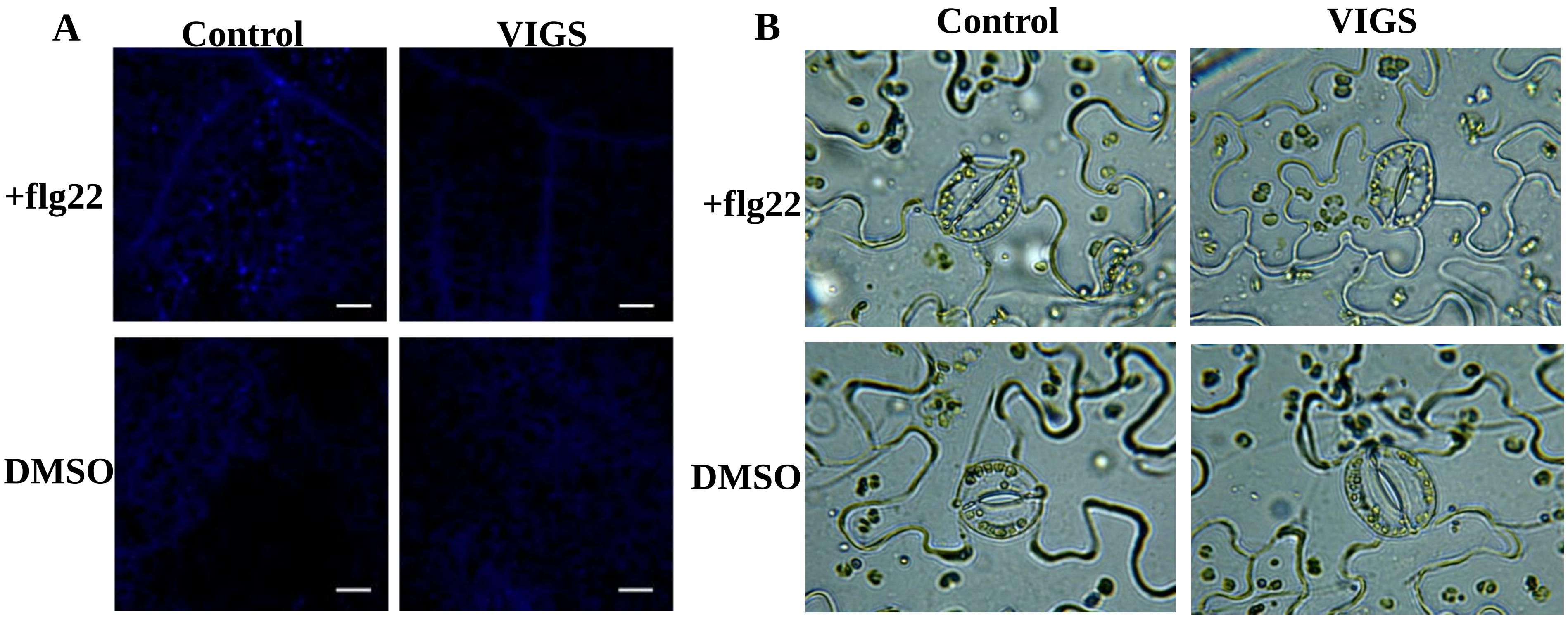

Figure S6 Characteristic of callose deposition and stomatal closure in *NbPLC2s*-silenced plant.

Effect of *NbPLC2s*-silencing on flg22-induced stomatal closure and callose deposition. Control and *NbPLC2s*-silenced (VIGS) leaves were treated with flg22 (+flg22) or DMSO (DMSO) solution. Observation of callose deposition (A) and stomatal closure (B) was carried out by the methods described in the materials and methods. Bar indicates 100 µm.

Table S1 List of primers used in this study.

Primers	Sequence	Purpose
M4	5’-GTTTTCCCAGTCACGAC-3’	Sequencing
RV	5’-CAGGAAACAGCTATGGAC-3’	Sequencing
PLC2s VIGSS	5’- GTCGACCATCTAACTCCAGATCTTCAGGC-3’	For VIGS vector
PLC2s VIGSA	5’- GTCGACCTTTCTTTGAGGCATCTTTTTCC-3’	For VIGS vector
PLC2-1 VIGSS	5’- GTCGACGGAAGGGAGAGAAGTACAG-3’	For VIGS vector
PLC2-1 VIGSA	5’- GTCGACATATTTCTCAACCATAACTCGAAC-3’	For VIGS vector
PLC2-2 VIGSS	5’- GTCGACGGAAGGGAGAGAAGTACAC-3’	For VIGS vector
PLC2-2 VIGSA	5’- GTCGACGGCAAAATATTTCTCAACTCG-3’	For VIGS vector
PLC2-1rtpF	5’- GGAGAAAAATGTAACTTTG-3’	qRT-PCR
PLC2-2rtpF	5’- GGAGAAAAATGTAACCTTA-3’	qRT-PCR
PLC2-1&2-2rtpR	5’- CCTGTGTATATGTAGTAATG-3’	qRT-PCR
NbAcre31rtp F	5’- AAGGTCCCGTCTTCGTCGGATCTTCG-3’	qRT-PCR
NbAcre31rtp R	5’- AAGAATTCGGCCATCGTGATCTTGGTC-3’	qRT-PCR
NbPti5rtp F	5’- CCTCCAAGTTTGAGCTCGGATAGT-3’	qRT-PCR
NbPti5rtp R	5’- CCAAGAAATTCTCCATGCACTCTGTC-3’	qRT-PCR
NbACTrtpF	5’-CGGAATCCACGAGACTACATAC-3’	qRT-PCR
NbACTrtpR	5’-GGGAAGCCAAGATAGAGC-3’	qRT-PCR
PR-4rtpF	5’-GGCCAAGATTCCTGTGGTAGAT-3’	qRT-PCR
PR-4rtpR	5’-CACTGTTGTTTGAGTTCCTGTTCCT-3’	qRT-PCR

Table S2 List of plasmids used in this study.

Plasmid	Relevant characteristics	Reference
pMD20	Cloning vector pMD20, Amp^r	Takara Bio
pMD-NbPLC2s	pMD20 carrying identical sequence of *NbPLC2-1 and NbPLC2-2* fragment for VIGS	This study
pMD-NbPLC2-1	pMD20 carrying *NbPLC2-1* fragment for VIGS	This study
pMD-NbPLC2-2	pMD20 carrying full length *NbPLC2-2* fragment for VIGS	This study
pPVX201	pPVX binary vector, Km^r	Baulcombe *et al.,* 1995
pPVXPLC2s	pPVX binary vector containing identical sequence of *NbPLC2-1 and NbPLC2-2* fragment for VIGS	This study
pPVXPLC2-1	pPVX binary vector containing PLC2 -1 cDNA fragment for VIGS	This study
pPVXPLC2-2	pPVX binary vector containing PLC2 -2 cDNA fragment for VIGS	This study
P35S-GUS	pGreen binary vector carrying *ß-glucronidase* (GUS)	Katou *et al.,* 2003
P35S-AvrA	pMDC32-derived plasmid that contains the full-length *avrA*	Poueymiro et al. 2009
P35S-PopP1	pAM-PAT-P35S-GW-CFP–derived plasmid that contains the *popP1*	Poueymiro et al. 2009
